# Amniotic fluid embolism: an Australian-New Zealand population-based study

**DOI:** 10.1186/s12884-015-0792-9

**Published:** 2015-12-24

**Authors:** Nolan McDonnell, Marian Knight, Michael J. Peek, David Ellwood, Caroline S. E. Homer, Claire McLintock, Geraldine Vaughan, Wendy Pollock, Zhuoyang Li, Nasrin Javid, Elizabeth Sullivan, on behalf of the Australasian Maternity Outcomes Surveillance System (AMOSS)

**Affiliations:** School of Women’s and Infants’ Health and School of Medicine and Pharmacology, University of Western Australia, Perth, Australia; National Perinatal Epidemiology Unit, University of Oxford, Oxford, UK; Sydney Medical School Nepean, The University of Sydney, Sydney, Australia; School of Medicine, Gold Coast Campus, Griffith University, Gold Coast, Australia; Gold Coast University Hospital, Gold Coast, Australia; University of Technology Sydney, Faculty of Health, Sydney, Australia; National Women’s Health, Auckland City Hospital, Auckland, New Zealand; Judith Lumley Research Centre, La Trobe University, Melbourne, Australia; Department of Nursing, Melbourne School of Health Sciences, The University of Melbourne, Melbourne, Australia; School of Women’s and Children’s Health, The University of New South Wales, Sydney, Australia; Department of Anaesthesia and Pain Medicine, King Edward Memorial Hospital, 374 Bagot Road, Subiaco, WA 6008 Australia; Department of Anaesthesia, St John of God Hospital (Subiaco), Perth, Australia

**Keywords:** Amniotic fluid embolism, Maternal mortality, Postpartum hemorrhage, Blood transfusion, Reproductive techniques, Assisted

## Abstract

**Background:**

Amniotic fluid embolism (AFE) is a major cause of direct maternal mortality in Australia and New Zealand. There has been no national population study of AFE in either country. The aim of this study was to estimate the incidence of amniotic fluid embolism in Australia and New Zealand and to describe risk factors, management, and perinatal outcomes.

**Methods:**

A population-based descriptive study using the Australasian Maternity Outcomes Surveillance System (AMOSS) carried out in 263 eligible sites (>50 births per year) covering an estimated 96 % of women giving birth in Australia and all 24 New Zealand maternity units (100 % of women giving birth in hospitals) between January 1 2010-December 31 2011. A case of AFE was defined either as a clinical diagnosis (acute hypotension or cardiac arrest, acute hypoxia and coagulopathy in the absence of any other potential explanation for the symptoms and signs observed) or as a post mortem diagnosis (presence of fetal squames/debris in the pulmonary circulation).

**Results:**

Thirty-three cases of AFE were reported from an estimated cohort of 613,731women giving birth, with an estimated incidence of 5.4 cases per 100 000 women giving birth (95 % CI 3.5 to 7.2 per 100 000). Two (6 %) events occurred at home whilst 46 % (*n* = 15) occurred in the birth suite and 46 % (*n* = 15) in the operating theatre (location not reported in one case). Fourteen women (42 %) underwent either an induction or augmentation of labour and 22 (67 %) underwent a caesarean section. Eight women (24 %) conceived using assisted reproduction technology.

Thirteen (42 %) women required cardiopulmonary resuscitation, 18 % (*n* = 6) had a hysterectomy and 85 % (*n* = 28) received a transfusion of blood or blood products. Twenty (61 %) were admitted to an Intensive Care Unit (ICU), eight (24 %) were admitted to a High Dependency Unit (HDU) and seven (21 %) were transferred to another hospital for further management. Five woman died (case fatality rate 15 %) giving an estimated maternal mortality rate due to AFE of 0.8 per 100 000 women giving birth (95 % CI 0.1 % to 1.5 %). There were two deaths among 36 infants.

**Conclusions:**

A coordinated emergency response requiring resource intense multi-disciplinary input is required in the management of women with AFE. Although the case fatality rate is lower than in previously published studies, high rates of hysterectomy, resuscitation, and admission to higher care settings reflect the significant morbidity associated with AFE. Active, ongoing surveillance to document the risk factors and short and long-term outcomes of women and their babies following AFE may be helpful to guide best practice, management, counselling and service planning. A potential link between AFE and assisted reproductive technology warrants further investigation.

## Background

Amniotic fluid embolism (AFE) is a rare and incompletely understood condition that is unique to pregnancy. The condition is characterised by the sudden onset of maternal compromise generally involving the cardio-respiratory and haematological systems, which can rapidly progress to cardiac arrest and profound coagulopathy leading to death [1]. Despite its rarity, AFE is now a leading cause of maternal mortality in a number of countries, and in Australia [2] and New Zealand [3] it is the leading cause of direct maternal mortality. The rarity of the condition and the lack of a reliable animal model hamper efforts to study the condition in greater depth, hence case registries and the publication of case reports are important in the advancement of knowledge of AFE. The Australasian Maternity Outcomes Surveillance System (AMOSS) was established across maternity units in Australia and New Zealand in 2009 in order to study rare and serious disorders of pregnancy [4, 5]. The aim of this paper was to estimate the incidence of AFE in Australia and New Zealand and to describe risk factors, management, and outcomes for women from the first two years of AMOSS surveillance into AFE.

## Methods

AMOSS is a bi-national surveillance and research system that utilizes a web based prospective clinical audit of a range of rare disorders of pregnancy. Ninety four percent of eligible Australian maternity units (*n* = 263/279) covering an estimated 96 % of women giving birth in hospital and all 24 New Zealand maternity units (100 % of hospital births) participate in AMOSS, with equal representation of public and private hospital sites.

A staggered implementation of AMOSS occurred as ethics, governance and site approvals were granted between 2008-2011 [4]. AMOSS sites were eligible for participation across Australia and New Zealand if they had over 50 births per year. At the commencement of surveillance in January 2010, 107 (37 %) of 291 sites were active, covering 59 % of births across Australia and New Zealand. This rose to 260 sites covering 90 % of births by December 2010 and to 291 of 301 eligible sites by December 2011 [5].

Women with AFE were identified by AMOSS data collectors (predominantly midwives, obstetricians, anaesthetists and health information managers) in maternity units through a monthly active negative surveillance system between January 1, 2010 and December 31, 2011. The average monthly response rate for active surveillance was 88 % during the study period. After data collectors notified a case, they were then prompted to confirm case eligibility by responding to a standardised case definition. A case of AFE was defined either as a clinical diagnosis (acute hypotension or cardiac arrest, acute hypoxia and coagulopathy in the absence of any other potential explanation for the symptoms and signs observed) or as a post mortem diagnosis (presence of fetal squames/debris in the pulmonary circulation). Sites were provided with clear guidance on the case definition and reporting criteria. In cases with any uncertainty over the diagnosis, they were able to contact the investigating team for guidance prior to submitting a case report.

Data were collected using two secure, web-based clinical data collection forms. The first form captured data on baseline demographic and pregnancy factors, obstetric interventions and perinatal outcomes and was utilised for all conditions being studied by AMOSS. The second form was a case-specific questionnaire, which included the location of the woman’s initial presentation/collapse; the most prominent initial presenting feature; other associated features; therapy required (including cardio-pulmonary resuscitation (CPR), intubation/ventilation, admission to ICU or HDU, blood and blood products); results of relevant investigations and maternal and neonatal outcomes. Data collectors were contacted regarding missing data or where data were not consistent with expected values to ensure validation. Free-text responses to questions regarding medical or obstetric morbidity were classified according to ICD-10-AM [6].

Ethics and governance processes for AMOSS ranged from a single application, multi-region ethics approval (MEC/09/73/EXP) in New Zealand, to multiple Human Research Ethics Committees for individual sites across Australia. Over 46 full ⁄ expedited ethics applications, 131 site governance applications and 136 letters of support requests were made over a 33 month period in Australia [4]. The current list of participating sites is listed in AMOSS newsletters [7]. Informed consent from women or their next of kin to participate in AMOSS was not required from the lead Human Ethics Committees (the New South Wales Population and Health Research Ethics Committee and the University of New South Wales Human Research Ethics Committee) or as part of the individual site approval processes.

### Statistical analyses

Incidence rates and maternal mortality ratios were calculated with 95 % confidence intervals (CI). Rates were calculated using denominators estimated from data provided by participating maternity sites on women giving birth (defined as the birth of one or more live or stillborn infants of at least 400 g birthweight, or at least 20 weeks’ gestation) in Australia and New Zealand. Denominator populations for Australia and New Zealand were estimated using the number of births in participating maternity sites between January 1 2010-December 31 2011. To address the staggered implementation of AMOSS, when defining birth rates, Australian birth denominators were adjusted for each hospital according to the number of days active as an AMOSS participating site. The incidence of AFE was calculated per 100,000 women giving birth.

## Results

Over the study period there were 33 reported cases of AFE associated with five maternal deaths (case fatality rate of 15 %). The five women who died were diagnosed on the basis of clinical criteria; three women who died did not have a post-mortem examination, and in the remaining two there was no evidence of fetal debris in the maternal circulation. Over the corresponding period in Australia and New Zealand there were 613,731 women giving birth in participating maternity sites, giving an incidence of 1 in 18,598, or 5.4 per 100,000 women giving birth (95 % CI 3.5–7.2 per 100,000) and a maternal mortality of 0.8 per 100,000 women giving birth (95 % CI 0.1-1.5 per 100,000).

The median age of women was 35 years (range 16–43) (Table 1). Eight women (24 %) had undergone a caesarean section in a previous pregnancy, five (15 %) had been diagnosed with a placenta praevia during the index pregnancy related to the AFE and four women (12 %) had episodes of antepartum haemorrhage. The index pregnancy was a multiple gestation in three women (9 %) and polyhydramnios was diagnosed in two women (6 %).

**Table 1:** Characteristics of women with Amniotic Fluid Embolism, Australia and New Zealand 2010–2011

Characteristic		N (%)
Age	<25	2 (6 %)
	25–29	6 (18 %)
	30–34	8 (24 %)
	>35	17 (52 %)
Gravidity	1	14 (42 %)
	2	6 (18 %)
	3 or more	13 (40 %)
Parity	0	17 (52 %)
	1	7 (21 %)
	2 or more	9 (27 %)
Previous Caesarean section		8 (24 %)
Assisted Conception		8 (24 %)
Multiple Pregnancy		3 (9 %)
Gestational age at birth	20 to 27 weeks	1 (3 %)
	28 to 31 weeks	1 (3 %)
	32 to 36 weeks	7 (21 %)
	37 to 41 weeks	24 (73 %)
Placenta Praevia		5 (15 %)
Antepartum Haemorrhage		4 (12 %)
Current smoker		3 (9 %)
BMI >35		2 (6 %)
Pre-eclampsia		2 (6 %)
Polyhydramnios		2 (6 %)
Gestational diabetes		2 (6 %)
Other hypertensive disorder		2 (6 %)

BMI: Body mass index

Data are number (percentage)

A total of 14 women (42 %) underwent either an induction or augmentation of labour [8 (24 %) induction only, 2 (6 %) augmentation only and 4 (12 %) both induction and augmentation] (Table 2). Fifteen events (46 %) occurred in the birth suite whilst 15 (46 %) occurred in the operating theatre, two events (6 %) occurred at home and in one woman the location was unknown (Table 3). One third (33 %) had a normal vaginal birth with the remainder having a caesarean section (67 %). The onset of the AFE episode was recorded as having occurred within 5 min before or after the birth in 48 % of women whilst overall 41 % occurred prior to birth and 57 % occurred post-partum (Table 3). In the women who were still pregnant at the time of the event, the maximum interval from the onset of the episode until birth was 89 min, whilst the latest presentation was recorded 240 min post-partum. Five women presented 30 min or more after delivery.

**Table 2:** Labour and mode of birth of women with Amniotic Fluid Embolism, Australia and New Zealand 2010–2011

		N (%)
Induction of labour	Yes	12 (36 %)
	No	9 (27 %)
	N/A (no labour)	12 (36 %)
Augmentation of labour	Yes	6 (18 %)
	No	15 (46 %)
	N/A (no labour)	12 (36 %)
Meconium stained liquor	Fresh	2 (6 %)
	None	30(91 %)
	Unknown	1 (3 %)
Method of birth		
	Vaginal birth	11 (33 %)
	Caesarean section	22 (67 %)
	Elective	6 (18 %)
	Non elective	8 (24 %)
	Not recorded	8 (24 %)

Data are number (percentage)

**Table 3:** Features of Amniotic Fluid Embolism at presentation, Australia and New Zealand 2010-2011

		Number of women experiencing feature (%)
Location of onset	Home	2 (6 %)
	Theatre	15 (46 %)
	Birth Suite	15 (46 %)
	Not recorded	1 (3 %)
Time point at onset of collapse	Pre-labour	5 (15 %)
	First stage of labour	4 (12 %)
	Second stage of labour	4 (12 %)
	Post partum	17 (52 %)
	Unknown/Not recorded	3 (9 %)
Initial presenting feature	Premonitory symptoms	9 (27 %)
	Hypotension	7 (21 %)
	Shortness of breath	5 (15 %)
	Acute fetal compromise	5 (15 %)
	Maternal haemorrhage	3 (9 %)
	Coagulopathy	3 (9 %)
	Cardiac arrest	2 (6 %)
	Abnormal cardiac rhythm	2 (6 %)
	Seizure(s)	1 (3 %)
Associated features	Maternal haemorrhage	26 (79 %)
	Hypotension	25 (76 %)
	Coagulopathy	24 (73 %)
	Premonitory symptoms	14 (42 %)
	Cardiac arrest	13 (39 %)
	Shortness of breath	12 (36 %)
	Acute fetal compromise	9 (27 %)
	Abnormal cardiac rhythm	8 (24 %)
	Seizure(s)	3 (9 %)

Data are number (percentage)

The most commonly reported initial presenting features were premonitory symptoms (27 %), hypotension (21 %), shortness of breath (15 %) and acute fetal compromise (15 %) (Table 3). Associated features included maternal haemorrhage (79 %), hypotension (76 %) and coagulopathy (73 %) (Table 3).

Cardiopulmonary resuscitation was required for 42 % of women (Table 4). Blood products were administered to the majority of women (Table 4) and included red cells (85 %), cryoprecipitate (73 %), fresh frozen plasma (73 %) and platelets (58 %). Six women (18 %) had a hysterectomy to control bleeding. Laboratory markers indicating a significant coagulopathy were frequently recorded with a fibrinogen of <2 g/dl in 64 %, a platelet count of <100 × 10^9^/L in 61 % and an INR >1.5 in 48 %. Twenty (61 %) women were admitted to ICU, eight (24 %) women to an obstetric HDU, and three women (9 %) died before being transferred to ICU or HDU for admission. Seven women (21 %) required transfer to another hospital for further management.

**Table 4:** Management of women with Amniotic Fluid Embolism, Australia and New Zealand 2010-2011

		Number of women (%)	
			Median (IQR)
Transfer to other hospital site		7 (21 %)	
Hysterectomy		6 (18 %)	
Plasmaphoresis		2 (6 %)	
Recombinant Factor VIIa		8 (24 %)	
Intubation/Ventilation		14 (42 %)	
ECMO		0 (0 %)	
CPR		14 (42 %)	
Blood and blood products	Yes	28 (85 %)	
	Red Cells	28 (85 %)	9.5 (5.3–13.8)
	Fresh Frozen Plasma	24 (73 %)	6.0 (4–8.75)
	Cyroprecipitate	24 (73 %)	9.0 (4.3–20)
	Platelets	19 (58 %)	3.0 (2.0–4.0)
Blood Results (Worst recorded)	Haemoglobin <7 g/dl	17 (52 %)	
	Fibrinogen <2 g/dl	21 (64 %)	
	Platelets <100 10^9^/L	20 (61 %)	
	INR >1.5	16 (48 %)	
Level of care required	Required admission to ICU	20 (61 %)	
	Required admission to HDU only	8 (24 %)	
	Died prior to accessing ICU/HDU	3 (9 %)	
	Did not require ICU or HDU care	1 (3 %)	

*ECMO* Extracorporeal Membrane Oxygenation, *CPR* Cardiopulmonary resuscitation, *ICU* Intensive Care Unit, *HDU* High Dependency Unit

Data are number (percentage) or median (interquartile range (25 %–75 %))

Of the five reported maternal deaths secondary to AFE, three of the women were born outside of Australia or New Zealand; the age range was between 23 and 35 years and there was one twin pregnancy. Four women laboured, of which one had an induction of labour, one was augmented and one was both induced and augmented. Four women underwent an unplanned caesarean section and one had a vacuum extraction. The AFE first manifested between 5 min prior to the birth up until 18 min post-partum and the time from the onset of the episode until the time of death ranged from between 35 and 108 min in four women, with one woman surviving in an ICU environment for approximately three weeks.

The 33 women gave birth to 36 babies including one stillbirth prior to the onset of labour and one neonatal death (neonatal mortality of 2.7 %) of an infant born with a congenital abnormality that was not compatible with life. Twenty three of the babies were male (64 %) and 25 (69 %) required admission to a Neonatal Intensive Care Unit (NICU). Fourteen babies were unborn at the onset of the AFE episode; compared to babies born prior to the onset of the AFE episode they had lower 5 min Apgar scores (Apgar score <7 in 67 % versus 0 %), a higher NICU admission rate (100 % versus 53 %) and a higher requirement for intubation and ventilation (*n* = 7/14 (50 %) versus *n* = 1/16 (6 %)).

## Discussion

This study represents the first prospectively collected data on women with both fatal and non-fatal AFE in the Australian and New Zealand population. In total, 33 cases of AFE were reported, giving an incidence of 5.4 per 100,000 women giving birth. There were five deaths secondary to AFE, a maternal mortality of 0.8 per 100,000 women giving birth. Although described as a rare condition, AFE has long been established as the leading cause of direct maternal mortality in Australia [2] as well as in New Zealand [3], occurring more frequently than maternal deaths from hypertensive disorders, sepsis, obstetric haemorrhage and venous thromboembolism. Internationally AFE ranks as a leading cause of maternal mortality in many developed countries, and in the UK triennial report AFE was ranked as the fourth leading cause of direct maternal mortality [8], with a comparable maternal mortality to that reported in this study of 0.57 per 100,000 women. AFE is a poorly understood condition and because of its rare and unpredictable nature, combined with the lack of an appropriate animal model to study the condition, the use of case registries and case series is required to further advance knowledge of the condition.

The incidence of AFE reported in our study appears higher than those reported from similarly high resource countries [9] but consistent with previously reported data from Australia [9]. The UK Obstetric Surveillance System (UKOSS) [10], using similar methodology, reported an incidence of AFE of 2.0 per 100,000 deliveries compared to the incidence of 5.5 per 100,000 reported here. A number of factors may influence the reported incidence of AFE. Firstly, as there is no specific diagnostic test for AFE, it remains a diagnosis of exclusion [11]. Even though there are generally consistent diagnostic criteria for AFE across the major registries, whether a woman is reported as experiencing AFE may still come down to clinical judgement. This is likely to result in both false positive and false negative cases occurring. In addition, with the increasing recognition of AFE as a potential cause of maternal compromise, it is likely that less severe cases are now being included in registries and surveillance systems [12].

How AFE cases are identified may also influence the reported incidence of the condition. Knight et al. recently showed that using population-based data to determine the incidence of AFE resulted in more than double the number of cases that were identified with a specific validated case identification system [9]. When additional criteria were used to exclude potential false positive cases, data more consistent with case validation systems were obtained. Currently, as has been previously recommended [9], the ideal approach to improve the accuracy of case identification may be case notification followed by a detailed review of the medical record by a recognised expert.

A potential limitation of this study is the impact of the incremental participation by Australian maternity units in AMOSS during the initial study period. The implementation of AMOSS across Australian units was staggered as governance and site approvals were granted between 2008 and 2011 [4]. This was necessary as there was no consistent process for ethical approval across all maternity units in Australia, compared to NZ which has a streamlined approval system for multicentre research [13]. In January 2010, at the commencement of surveillance, 107 (37 %) of 291 sites were active, covering 59 % of births across Australia and New Zealand rising to 291(97 %) of 301 eligible sites by December 2011. In addition, this study focused on the initial presentation and management of the AFE. Longer term data in relation to outcomes such as neurological deficits was not able to be captured. With the de-identified nature of the data entry there was no ability to cross reference the maternal deaths that occurred with the currently available maternal mortality reporting systems in Australia and New Zealand. With the rare nature of the condition, cases which may have been missed because of presentation to a non obstetric hospital may have significant impact on the incidence and outcome data.

The results presented in this study confirm that management of women with AFE requires significant utilisation of resources such as operating theatre, transfusion medicine and intensive care services. AFE is unpredictable and may be associated with rapid maternal compromise. Health and maternity care providers, either in the community or hospital setting, must be aware of the key differences in the resuscitation of pregnant compared to non pregnant women [14]. Particular emphasis on the need for relief of aortocaval compression and the role of peri-mortem caesarean section in maternal resuscitation is important [15]. Previous maternal mortality reports have shown that maternal resuscitation is often substandard and emphasise the importance of training courses for staff in obstetric emergencies [8, 16]. Of importance, a collapse secondary to AFE may occur in any delivery setting, reinforcing the need for all health care providers who may care for pregnant women to be adequately trained in their initial resuscitation. It is encouraging that in this study, women who did suffer a cardiac arrest still had a high survival rate. In total 14 women underwent CPR for a presumed cardiac arrest and at least 9 (64 %) of these women survived. The high survival from cardiac arrest is likely to be multifactorial in nature and a reflection of the comparatively younger age compared to other patients who may suffer a cardiac arrest, lack of underlying co-morbidities such as ischaemic heart disease, increased physiological reserve associated with pregnancy in addition to the presentation likely occurring in environments with close monitoring and access to emergency care.

In women who survive the initial cardiopulmonary compromise, management of coagulopathy and major obstetric haemorrhage assume critical importance. Peri-partum hysterectomy is frequently required to control bleeding and a significant number of women will require admission to intensive or high dependency care services, as well as increased levels of care for their newborn. Not all centres that care for pregnant women may have onsite access to these facilities, especially in rural or remote areas and this study highlights the importance for all centres to have procedures to ensure timely transfer of women and their babies to appropriate centres.

The mortality associated with AFE was initially reported to be very high, with case fatality rates of 67-86 % reported in some of the first case series of AFE [17, 18]. The case fatality rate of 15 % reported here is consistent with previous reports from Australia [9], the UK [10], Netherlands [19], Canada [20] and the USA [21]. Factors that may contribute to a lower case fatality rates include an improved awareness of the condition so that women with less severe AFE are now included in registries, in addition to improvements in the resuscitation of pregnant women and advances in intensive care. The cornerstone of management is early recognition of the condition with prompt supportive measures. Premonitory symptoms, shortness of breath, hypotension and acute fetal compromise are consistently the earliest indications of AFE and should not be ignored.

A number of risk factors have been previously identified for AFE including advanced maternal age, polyhydramnios, multiple gestation, placental abnormalities and caesarean section [9, 21]. Whilst the numbers reported in this study are comparatively small, these risk factors would appear to be consistent when the women reported in this study are compared to the overall maternity population in Australia over a similar time period [22]. In a group of control women identified from other AMOSS studies, 24.5 % were aged 35 or over, 2.4 % had a multiple pregnancy and 19.3 % a previous caesarean delivery [23]. Induction and/or augmentation of labour has previously been suggested as a potential risk factor for AFE [9, 20, 24], in this study 36 % of women were classified as having an induction of labour whilst over a similar period of time population based Australian data showed that the overall induction rate was 26 % [22]. Whilst this supports the potential link between AFE and induction of labour, the small numbers reported here mean that this should be interpreted with caution. There were a high number of women who had had assisted conception in our study (8/33, 24 % vs national figures of 3.8 % [22]), to our knowledge the use of assisted reproductive technology has not been previously examined as a potential risk factor for AFE. Whilst the higher incidence may potentially be explained by the relationship to other established risk factors such as advanced maternal age, given the potential immune mediated theories for the pathogenesis of AFE this finding warrants further investigation.

The neonatal outcomes reported in this study compare favourably to previously reported data and are notable for a low stillbirth and neonatal death rate. A number of studies have reported high stillbirth and neonatal death rates [9, 19], whilst other studies have reported few or no neonatal deaths [20]. This is a welcome finding even noting the limitation of the small number of cases. This study highlights that the neonatal outcomes associated with AFE are likely related to the birth status of the neonate at the time of the onset of the AFE episode. Those babies who were born prior to the onset of the episode were born in much better condition then those born after the onset of the AFE, with better 5 min Apgar scores and less need for NICU admission and invasive ventilation. Fetal heart rate abnormalities are a common presenting feature of AFE, highlighting the sensitivity of the fetus to any compromise of the maternal state. Appropriate resuscitation of the mother, including the performance of a peri-mortem caesarean section if indicated, is an important component of the management process.

## Conclusion

This study shows that the majority of women with AFE suffer significant morbidity but survive and highlights the importance a heightened awareness of this condition by clinical staff caring for pregnant women along with the need to maintain competence in resuscitation. Ongoing surveillance to document the risk factors and short and long-term outcomes of women and their babies following AFE may be helpful to guide best practice, management, counselling and service planning. A potential link between AFE and assisted reproductive technology warrants further investigation.

## Abbreviations

AFE: Amniotic fluid embolism
AMOSS: Australasian Maternity Outcomes Surveillance System
BMI: Body mass index
CPR: Cardiopulmonary resuscitation
ECMO: Extracorporeal membrane oxygenation
HDU: High dependency unit
ICU: Intensive care unit
NICU: Neonatal intensive care unit
UKOSS: United Kingdom obstetric surveillance system
